# Newborn Screening for Spinal Muscular Atrophy in the UK: Use of Modelling to Identify Priorities for Ongoing Evaluation

**DOI:** 10.3390/ijns12010003

**Published:** 2026-01-13

**Authors:** Praveen Thokala, Alice Bessey, Rachel Knowles, John Marshall, Cristina Visintin, Miranda Lawton, Silvia Lombardo

**Affiliations:** 1 PT Health Economics Ltd., Sheffield S7 1RH, UK; 2 Sheffield Centre for Health and Related Research (SCHARR), University of Sheffield, Regent Court, 30 Regent Street, Sheffield S1 4DA, UK; 3 Population, Policy and Practice Research and Teaching Department, Great Ormond Street Institute of Child Health, University College London, 30 Guilford Street, London WC1N 1EH, UK; rachel.knowles@ucl.ac.uk; 4 UK National Screening Committee Secretariat, Department of Health and Social Care, 39 Victoria Street, London SW1H 0EU, UK; john.marshall@dhsc.gov.uk (J.M.); cristina.visintin@dhsc.gov.uk (C.V.); miranda.lawton@dhsc.gov.uk (M.L.); silvia.lombardo@dhsc.gov.uk (S.L.)

**Keywords:** newborn screening, spinal muscular atrophy, clinical and cost-effectiveness model, in-service evaluation

## Abstract

Spinal muscular atrophy (SMA) is a genetic condition that causes the degeneration of motor neurons in the spinal cord. Newborn blood spot (NBS) screening can potentially enable diagnosis before symptoms, and presymptomatic treatment is considered to be more effective than symptomatic treatment. In this paper, we present an overview of a cost-effectiveness model of NBS screening for SMA in the UK, informed by key clinical trials and the relevant published literature. Our analyses suggest that implementing screening could result in better outcomes and lower costs compared to the current approach of no screening plus treatment. However, several uncertainties and limitations of the model remain. These include uncertainty in the reimbursement status of nusinersen and risdiplam in the future; the ‘actual’ costs of treatments, as they are under confidential commercial agreements; uncertainty in the long-term effectiveness of presymptomatic and symptomatic treatment; and uncertainty around the incidence of SMA and the costs and the accuracy of NBS screening. An SMA in-service evaluation (ISE) that could capture data specific to the UK is under consideration, and an appropriately designed ISE with ongoing data collection could support periodic updates of clinical and cost-effectiveness estimates of NBS screening for SMA in the UK.

## 1. Introduction

Spinal muscular atrophy (SMA) is an autosomal recessive disease involving degeneration of the alpha motor neurons in the spinal cord, resulting in symmetrical muscle weakness and atrophy, with the impact upon the muscles used to support breathing leading to lethal consequences. SMA is traditionally categorised into five different types according to the age of symptom presentation and diagnosis, from type 0 (the most severe, identified at birth) to type 4 (becoming symptomatic in adulthood and usually constituting mild disease). The vast majority of SMA cases (95%) are due to a homozygous deletion in the survival motor neuron 1 (*SMN1*) gene, which leads to a decrease in the functional SMN protein and ultimately leads to patients developing SMA. The related survival motor neuron 2 (*SMN2*) gene can also produce SMN protein, but only approximately 10% of the SMN protein coming from this route is functional [1,2].

There are now three treatments available for patients with SMA: nusinersen (Spinraza) and risdiplam (Evrysidi) are recurrent treatments, while onasemnogene abeparvovec (Zolgensma) is a one-off gene therapy. The national criteria for treatment in England are determined by the National Institute for Health and Care Excellence (NICE). From the limited evidence that is available, it appears that presymptomatic treatment is more effective than symptomatic treatment, and newborn blood spot (NBS) screening potentially allows babies to be diagnosed before they show signs or symptoms [3,4,5,6]. Decision making on treatment options after NBS screening is based on presymptomatic diagnosis using *SMN2* copy numbers, with a higher number of *SMN2* copies generally correlating with reduced disease severity but imperfectly correlating with clinically defined SMA types [7].

In the four UK countries, the UK National Screening Committee (UK NSC) advises ministers and the National Health Service (NHS) about all aspects of screening, including the case for introducing new conditions to the NHS NBS Screening Programme [8,9]. The UK NSC also maintains oversight of the evidence relating to the balance of good and harm and the impact on healthcare costs and health consequences of existing, new, or modified screening programmes [9]. Decision-analytic models offer a structured framework to simulate the potential pathways of care for different strategies of screening programmes compared with no screening and a mechanism to bring together all available, relevant evidence on effectiveness, harms, health benefits, and healthcare costs. Decision-analytic modelling to estimate cost-effectiveness (also referred to as ‘modelling studies’) is increasingly being used in the development of UK NSC recommendations on NBS screening [10]. Modelling studies can usefully address the absence of clinical trial evidence comparing screening with no screening or current practice. However, modelling studies are based on data from different sources and are constrained by a range of issues that are intrinsic to the rare disease setting. These include small sample sizes of clinical studies; lack of head-to-head data on treatment options; uncertainty in the benefits and harms conceptualised in NBS screening models; and limited availability of long-term data relating to the modelled strategies. These limitations create uncertainty about both clinical and cost-effectiveness results and their generalisability to UK healthcare jurisdictions [10,11,12,13].

In the UK, the UK NSC makes use of in-service evaluation (ISE) activity to test a proposal for a new screening programme or a change to an existing one within ‘real world’ NHS service delivery. The primary aim of ISE is to deliver robust evidence, generated in an NHS setting, to support a UK NSC recommendation to the four UK nations. When the remaining evidence gaps can only feasibly be filled by large-scale work in live NHS services, then the UK NSC can recommend ISE work [14]. Recently, an ISE for newborn screening for severe combined immunodeficiency (SCID) was completed in England [15,16], and another one in the cancer screening setting has also recently been commissioned [17]. There are several uncertainties that specifically relate to estimating the clinical and cost-effectiveness of NBS screening for SMA in the UK. These include uncertainty in accurately predicting severity or type of SMA from genetic information alone, reimbursement status of symptomatic and presymptomatic treatments for SMA currently in England in the NHS (NICE is currently appraising nusinersen and risdiplam for symptomatic and presymptomatic treatment of SMA with the recommendations scheduled for early 2026 [6]), and the cost of treatments (all the treatments for SMA are under confidential patient access schemes in the NHS, and as such, the “actual” prices of these treatments are unknown).

A ‘flexible’ model, commissioned by the UK NSC, was developed to address these uncertainties. This paper presents a brief overview of the model and describes how the modelling results assisted in identifying priorities for a future ISE of NBS screening for SMA.

## 2. Methods

The model structure and inputs were developed through findings from cost-effectiveness models of NBS screening for SMA [18,19,20,21,22,23,24,25,26], and reviews on presymptomatic treatment for SMA and accuracy of newborn screening for SMA [27,28]. In addition, 4 online workshops with key experts were conducted between September 2023 and November 2024, with around 20 participants in each workshop. The workshops elicited expert input to finalise the model assumptions, to identify the best sources of data for populating the model, to address key uncertainties in the modelling and input data, and to identify any changes needed following presentation of the preliminary results.

### 2.1. Model Overview

**Figure 1:**
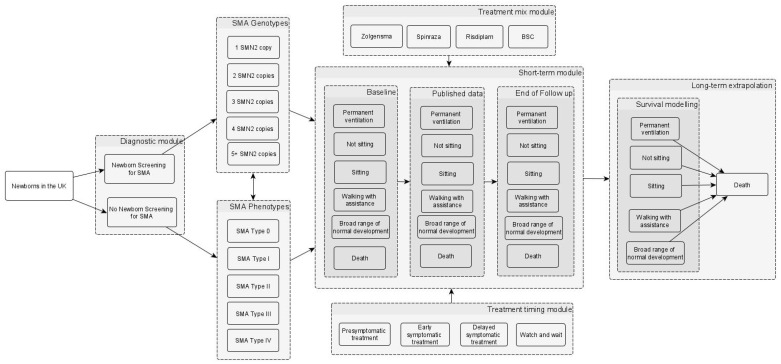
Simplified model structure of NBS screening for SMA. The model begins with the population (a hypothetical cohort of newborns in the UK). The population in the no NBS screening arm is the same as in the NBS screening arm in terms of the incidence of SMA and the proportions of different genotypes and phenotypes (i.e., the different SMA types). The box labelled “short-term module” represents a 3-year short-term model which incorporates the motor function milestones gained (i.e., sitting, walking with assistance, and broad range of normal development), the need for permanent ventilation, the time to death, and the treatment effectiveness based on clinical study data. In the short-term module box, the “baseline”, “published data”, and “end of follow-up” in the different columns relate to the 6-monthly time intervals, where data on the proportions of patients in the different health states are sourced from the key clinical studies of the different treatments. The long-term model on the right involves the extrapolation of the motor function milestones, the need for permanent ventilation, and mortality, which are assumed to be conditional on the health states reached by the end of the 3-year model. The long-term model uses 6-monthly time cycles to estimate the lifetime costs and quality-adjusted life years. Although not depicted in the figure, a BSC scenario was also included as a comparator. Abbreviations: BSC: best supportive care; NBS: newborn blood spot; SMA: spinal muscular atrophy; SMN: survival motor neuron. Source: [40].

A model was developed to estimate the clinical and cost-effectiveness of NBS screening for SMA, informed by key clinical trials and the relevant published literature [29,30,31,32,33,34,35,36,37]. As shown in Figure 1, the model uses a decision tree (for the screening phase), followed by a 3-year short-term model (for incorporating treatment effectiveness based on clinical study data) and long-term modelling (for extrapolation based on survival modelling) using a six-month model cycle. A best supportive care (BSC) arm (BSC refers to symptom management/watch and wait for cases of milder disease or it refers to palliative type care for patients with SMA type 0 and those with very severe disease) was also included as a comparator to explore the magnitude of clinical effect of the three treatments in settings with and without screening, similar to the approach used in other evaluations [38,39]. The aim of including BSC was to provide further perspective on the improvement of outcomes from SMA within these respective settings. While the comparator for cost-effectiveness was ‘no screening’ (i.e., use of the three treatments in clinically diagnosed SMA), the BSC arm provided security against the uncertainty relating to the status of two of the drugs in the NICE evaluation process. For example, if risdiplam and/or nusinersen are not approved for routine use in the NHS by NICE [6], it will be important to capture the untreated groups as part of both the no screening and screening arms in future iterations of the model.

### 2.2. Model Inputs

The model inputs are sourced from the relevant published literature and supplemented with expert opinion where necessary, as shown in Table 1. A brief overview of the model inputs is presented here, and a detailed description of the model inputs, sources, and assumptions is presented in the Supplementary Material (File S1).

The epidemiological data includes the incidence of SMA and the proportions of different genotypes, with the mapping between genotypes and phenotypes ensuring that the population in the no NBS screening arm is the same as in the NBS screening arm.

The treatment effectiveness is based on the proportions of patients receiving the different treatments (zolgensma, nusinersen, risdiplam, or BSC), either presymptomatically or symptomatically, according to the number of *SMN2* copies (1, 2, 3, 4, 5+) in the NBS screening arm and the different SMA types (0, 1, 2, 3, 4) in the no NBS screening arm.

Data on motor function milestones, permanent ventilation, and mortality over different time points were extracted from the relevant trials/studies and additional follow-up data from registry data [29,30,31,32,33,34,35,36,37].

Long-term survival is assumed conditional on health states, so that each model health state (permanent ventilation (PV), not sitting, sitting, walking with assistance, and broad range of normal development (BRND)) has a mortality risk, and the impact of SMA symptoms on the patient is modelled using health state-specific costs and utilities.

The costs of NBS screening, costs of confirmatory tests, costs of symptomatic diagnosis, and costs of treatments were sourced from the published literature and expert opinion.

### 2.3. Model Analyses

The base case analysis in the model used an NHS and personal social services perspective, using the mean values of parameters to estimate cost-effectiveness [i.e., cost per quality-adjusted life-year (QALY)] results. To account for non-linearities amongst the model inputs, probabilistic sensitivity analysis (PSA) was undertaken using appropriate distributions to represent the uncertainty in the data inputs. The treatment prices used in the model are those publicly reported (list prices—see Table S16 in Supplementary Material, File S1), as the actual prices paid by the NHS are confidential. Sensitivity analyses assuming discounted prices were undertaken as the NHS is sometimes able to negotiate confidential discounts for individual drugs. The base case analysis included all available treatments at list price, and another analysis assuming a 30% discount for Zolgensma and a 90% discount for risdiplam and nusinersen. Zolgensma is a one-off treatment, so a lower discount was assumed, while the other two drugs have to be administered through the patient’s lifetime, so a greater discount was assumed. Scenario analyses were also performed using Zolgensma only, using list price and price discount, as presented elsewhere [40].

## 3. Results

### 3.1. Short-Term Outcomes

Table 2 provides an overview of the proportion of SMA cases identified symptomatically and presymptomatically in the different arms of the model. Using an annual cohort of 600,000 newborns in the UK, and an incidence rate of 1 in 8200 for SMA, results in 73.17 cases of SMA. In the BSC arm, it was assumed that all 73.17 cases of SMA would be diagnosed symptomatically and would receive BSC. In the No NBS screening arm of the model, 0.73 cases of SMA were detected presymptomatically via family history, with the remaining 72.44 cases detected symptomatically. In the NBS screening arm of the model, most of the SMA cases (*n* = 69.44) were detected presymptomatically, with the remaining 3.73 cases detected symptomatically (i.e., the 5% of patients who do not have homozygous deletions in *SMN1*).

### 3.2. Mid-Term Outcomes (Outcomes at the End of 3 Years)

**Figure 2:**
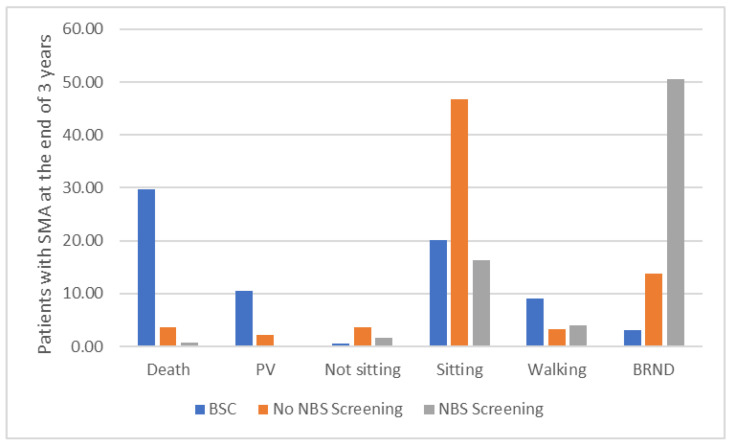
Patients at the end of 3 years in the BSC arm, the No NBS screening arm, and the NBS screening arm. The figure shows that, at the end of the 3 years, in the BSC arm, as the patients are detected symptomatically and only receive BSC (i.e., no pharmacological treatment), there are many deaths and patients on permanent ventilation. In the No NBS screening arm, where patients are detected symptomatically and receive pharmacological treatment, the number of deaths and patients on permanent ventilation are reduced substantially, but most of the patients are in the sitting health state, with only a few patients achieving walking with assistance or broad range of normal development. In the NBS screening arm, where most patients receive pharmacological treatment presymptomatically, the number of deaths and patients on permanent ventilation and patients in a sitting health state are further reduced, with most of the patients achieving broad range of normal development. Abbreviations: BRND: broad range of normal development; BSC: best supportive care; NBS: newborn blood spot; PV: permanent ventilation.

Figure 2 presents the comparison between the BSC arm, the No NBS screening arm, and the NBS screening arm at the end of the 3 years. This suggests that compared with the current practice (i.e., assuming all three drugs are available), NBS screening would prevent 2 cases requiring permanent ventilation, around three early deaths, and about 30 cases who do not progress beyond a sitting state. NBS screening also enables about 37 more cases who experience few or no significant limitations. However, NBS screening will identify around three cases with five *SMN2* copies who will not experience symptoms until adulthood, if at all, and identification may even be detrimental to their health and wellbeing. In the BSC arm, as the patients are detected symptomatically and only receive BSC (i.e., no pharmacological treatment), the outcomes at the end of the 3 years suggest that there are many deaths (29.69) and patients on permanent ventilation (10.54).

### 3.3. Long-Term Outcomes

**Figure 3:**
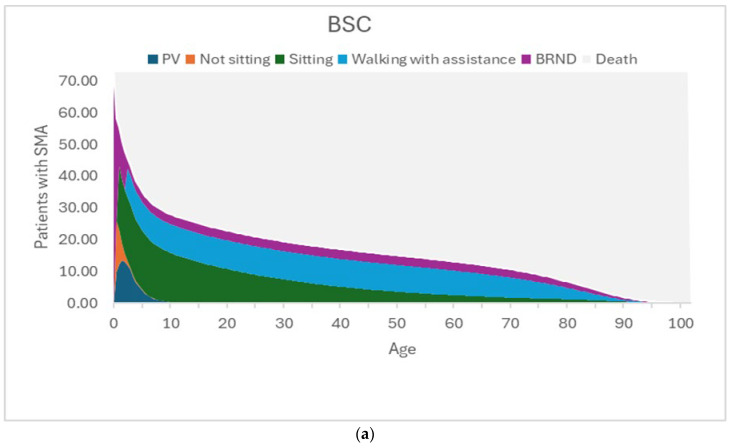

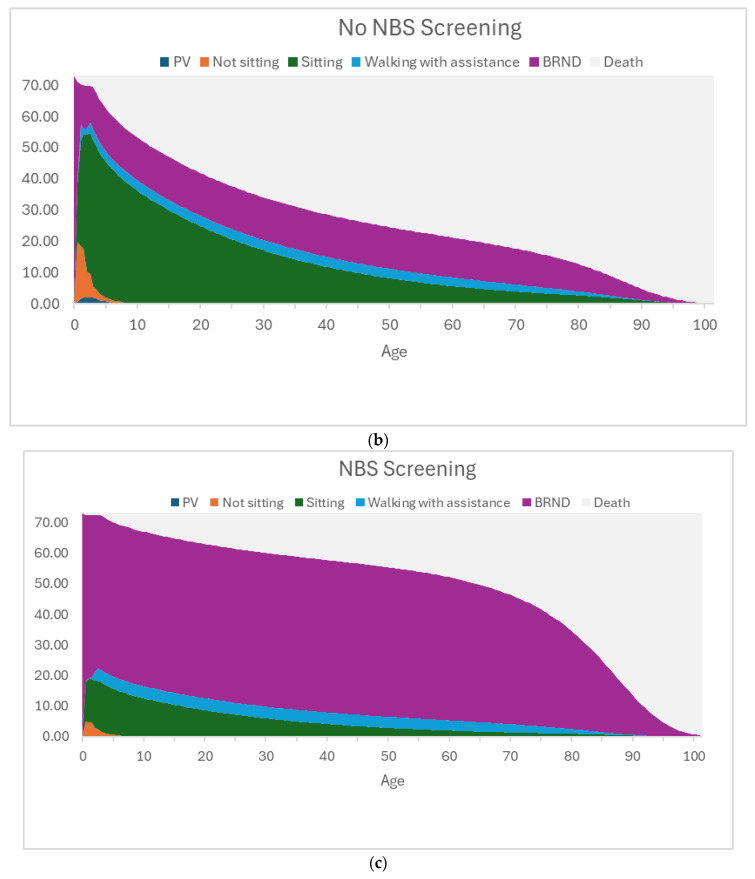
(**a**) Graphical representation of the time spent in each health state for the best supportive care arm. The figure shows the time spent in the different health states by the 73.17 patients with SMA. In this BSC arm, there are many deaths and patients on PV, with some patients in the sitting health state and a few in the walking with assistance health state. The lower survival of patients in the permanent ventilation and sitting health states is reflected in the long-term outcomes of patients in BSC. (**b**) Graphical representation of the time spent in each health state for the No NBS screening arm. The figure shows the time spent in the different health states by the 73.17 patients with SMA. In this No NBS screening arm, most of the patients are in the sitting health state and some are in the BRND state, and only a few patients are in the walking with assistance state. The survival in the No NBS screening arm is higher than that in the BSC arm due to lower short-term deaths and fewer patients in PV, but the lower survival of patients in sitting health states is reflected in the long-term outcomes of patients in the No NBS screening arm. (**c**) Graphical representation of the time spent in each health state for the NBS screening arm. The figure shows the time spent in the different health states by the 73.17 patients with SMA. In this NBS screening arm, the number of deaths and patients on PV and patients in the sitting health state are further reduced, with most of the patients achieving BRND. The survival in the NBS screening arm is much higher than that in the No NBS screening arm due to lower short-term deaths, fewer patients in PV, and fewer patients in sitting health states. Most of the patients in the NBS screening arm are in the BRND state (which assumes survival of the general population), and this is reflected in better long-term outcomes of patients in the NBS screening arm. Abbreviations: BRND: broad range of normal development; BSC: best supportive care; NBS: newborn blood spot; PV: permanent ventilation. Source: [40].

The model assumes that the motor function milestones achieved at the end of the 3-year short-term model are sustained until death, and the mortality in the long-term is modelled using survival curves for each of the motor function milestones based on data from the published literature [46,47]. Figure 3a–c present the time spent in different health states in the BSC arm, the No NBS screening arm, and the NBS screening arm.

In the BSC arm, the majority of patients die or are receiving PV, with smaller numbers of patients limited to the sitting health state, and a few achieving the walking with assistance and BRND health states. The lower survival of patients in PV and sitting health states is reflected in the long-term outcomes of patients in this BSC arm.

In the No NBS screening arm, most of the patients are in the sitting health state, and some are in the BRND state, and only a few patients are in the walking with assistance state. The lower survival of patients in sitting health states is reflected in the long-term outcomes of patients in the No NBS screening arm.

In the NBS screening arm, the number of deaths and patients on PV and patients in a sitting health state are reduced, with most of the patients achieving BRND. The survival in the NBS screening arm is much higher than that in the BSC and No NBS screening arms due to fewer early deaths, fewer patients in PV, and fewer patients in sitting health states. Most of the patients in the NBS screening arm are in the BRND state (whose survival is similar to that of the general population), and this is reflected in better long-term outcomes of patients in the NBS screening arm.

### 3.4. Cost-Effectiveness Results

Table 3 presents the cost-effectiveness results of the base case analysis, i.e., using all the treatments currently eligible (see report for the treatment mix describing the proportions of patients receiving the different treatments [40]) and using list prices and treatments assuming price discounts. Table 3 suggests higher total QALYs and lower total costs in the NBS screening arm compared to the No NBS screening arm, resulting in NBS screening dominating the No NBS screening arm. When using list prices, PSA results presented in the Supplementary Material (File S2) suggest that, at a threshold of GBP 20,000/QALY, NBS screening has a 90% probability of being cost-effective compared to No NBS screening. However, it should be noted that No NBS screening is not cost-effective compared to BSC, with an incremental cost-effectiveness ratio (ICER) of GBP 606,516/QALY. The ICER of NBS screening compared to BSC is GBP 219,393/QALY, suggesting that NBS screening is not cost-effective when compared to BSC at the typical NICE thresholds of GBP 20,000 to GBP 30,000/QALY.

Table 3 also presents the cost-effectiveness results of the analysis using a 30% discount for Zolgensma and a 90% discount for risdiplam and nusinersen. When using price discounts, NBS screening is dominating the No NBS screening arm, and the PSA results presented in Supplementary Material (File S2) suggest 100% probability of NBS screening being cost-effective compared to No NBS screening at thresholds greater than GBP 20,000/QALY. However, it should be noted that No NBS screening is not cost-effective compared to BSC with an ICER of GBP 210,930/QALY. The ICER of NBS screening compared to BSC is GBP 62,217/QALY, which could be considered cost-effective at thresholds of GBP 100,000/QALY used for NICE highly specialised technologies (HSTs) but not cost-effective at the typical NICE thresholds of GBP 20,000 to GBP 30,000/QALY.

## 4. Discussion

A model was developed to estimate the clinical and cost-effectiveness of NBS for SMA, informed by key clinical trials and the relevant published literature. However, there are several key uncertainties and limitations of the model. NICE is currently appraising nusinersen and risdiplam for symptomatic and presymptomatic treatment of SMA, with the recommendations scheduled for November 2025. As such, there is substantial uncertainty in the reimbursement status of the treatments in the future. Also, the costs of treatments are under confidential patient access schemes in the NHS, and as such, the “actual” prices of these treatments are unknown. There is also uncertainty in the effectiveness of presymptomatic and symptomatic treatment, with limited data on longer-term outcomes [3,4,5,6]. In addition, there is uncertainty in terms of the impact of diagnostic delay during the screening process on the number of patients becoming symptomatic with Type 1 SMA, and subsequently, the impact on outcomes achieved. There is also uncertainty around the incidence of SMA, and the cost, and the accuracy of NBS screening for SMA.

A Health Technology Assessment (HTA) commissioning brief for an ISE for SMA that would compare screened with unscreened populations within England is currently underway to address these uncertainties and provide UK-specific data [55]. Priorities identified for this ISE as part of the modelling work were: epidemiology, feasibility of real-world implementation, including clinical pathway, test methodology and accuracy, screening programme performance, acceptability of screening, and data on treatment effectiveness (particularly longer-term outcomes) and health state costs. Through real-life implementation in live NBS screening services for SMA, an appropriately designed ISE could provide more information on the epidemiology of SMA (i.e., data on incidence, breakdown of SMA types, and copy numbers) and the accuracy and costs of the NBS screening. The model assumed 99.9% sensitivity and specificity for the initial PCR test (excluding other variants, i.e., the 5% of patients who do not have homozygous deletions in *SMN1*), and the costs were based on expert opinion and a previous SCID evaluation [15,51]. As such, the ISE could provide more information about the clinical pathway and the associated costs/implications of NBS screening.

An ISE could also evaluate the timescales that can be met in UK services at important stages of the screening pathway, including when screening results become available, the timing of clinical referral, and starting treatment, if offered and accepted. The model assumed that around half of the patients diagnosed with two *SMN2* copies would show symptoms by treatment start, and these patients would receive early symptomatic treatment, which is likely to be less effective than presymptomatic treatment. An ISE could also address this uncertainty with real-world data on diagnostic and treatment delay, and the effectiveness of early symptomatic treatment.

Expert opinion was solicited to specify the treatment mix in the model (i.e., the proportions of patients receiving the different treatments, according to the different *SMN2* copies in the NBS screening arm and SMA types in the no NBS screening arm), and published data from pivotal trials was used to estimate the treatment effectiveness [29,30,31,32,33,34,35,36,37]. By the time that the ISE begins, NICE will have made recommendations about treatments (currently scheduled for early 2026); therefore, the ISE could provide information on which treatments patients receive in practice, the effectiveness of treatment for presymptomatic and asymptomatic patients, and the likelihood of patients becoming symptomatic due to delays in diagnosis. As such, an ISE could provide information on what treatments patients receive in practice and the treatment effectiveness in presymptomatic and early symptomatic treatment. A number of studies have been published in recent years [56,57,58,59], but uncertainties still remain. Higher quality evidence relies on a robust comparator and, in this rare disease setting, discussion about design options may need to consider the combination of prospective and retrospective datasets to improve the reliability of modelled comparisons.

As the treatments are quite new, further data on the clinical effectiveness in terms of the motor function milestones (i.e., sitting or walking with assistance and BRND) of treating presymptomatic babies is needed, and this could be provided by an ISE. Data on short-term outcomes will contribute evidence on the clinical effectiveness of screening at different time points (e.g., 1 year, 2 years, and 3 years). However, data on longer-term outcomes (e.g., 5 years and beyond) is an equally, if not more, important outcome. Despite the uncertainty on cost, it is assumed that the treatments are expensive. Understanding the clinical value over time is a key issue relating to economic investment, but also for clinicians who need to set realistic expectations for parents of affected babies who may be concerned, for example, about waning treatment effectiveness.

The model used published data for the health state costs and utilities to account for the impact of NBS screening for SMA symptoms on the patient, and the data from an ISE can provide more direct information regarding the quality of life and costs of the different health states. The recent SCID ISE [15,16] provides an example of the way in which data collection on these and other issues improved the quality of the modelled analysis of screening to inform UK NSC discussion. This might provide a useful point of reference for the design of an SMA ISE.

As the costs of treatments are under confidential patient access schemes, the list prices for the treatment costs were used in the base case analyses of the model, and sensitivity analyses were performed using hypothetical discounts. It is not clear whether an ISE would have access to this confidential pricing data, and as such, there is still uncertainty regarding the treatment costs to the NHS.

## 5. Conclusions

The model was populated using the best available data from the published literature, but key uncertainties and limitations remain. However, as outlined in this paper, an SMA ISE is under consideration, which could capture data specific to the UK. An appropriately designed ISE and ongoing data collection could support updated, periodic estimates of clinical and cost-effectiveness of NBS screening for SMA in the UK.

## Figures and Tables

**Table 1:** Summary of model inputs and sources.

Type of Parameter	Source
***Epidemiology***	
Incidence of SMA	Dangouloff et al. 2021 [41]
Proportions of *SMN2* copies	Muller-Felber et al. 2023 [42] Vill et al. 2021 [43] Boemer et al. 2021 [44] Wallace et al. 2023 [45]
Mapping between *SMN2* copies and SMA types	Calucho et al. 2018 [7]
***Treatment mix***	
Treatment by type of SMA	Expert opinion
Treatment by *SMN2* copy number	Expert opinion
***Treatment effectiveness***	
Presymptomatic treatment effectiveness	Strauss et al. 2022a [29] Strauss et al. 2022b [36] Servais et al. 2024 [30] Crawford et al. 2023 [31]
Symptomatic treatment effectiveness	Masson et al. 2021 [37] Deconinck et al. 2022 [32] Castro et al. 2020 [33] Mercuri et al. 2018 [34] Darras et al. 2019 [35]
Early symptomatic treatment effectiveness	Assumption
***Inputs for long-term modelling***	
Survival by health state	Gregoretti et al. 2013 [46] Zerres and Schoneborn et al. 1997 [47]
Utility by health state	López-Bastida et al. 2017 [48] Tappenden et al. 2018 [49] Landfelt et al. 2019 [50]
***Cost inputs***	
Cost related to newborn screening	Expert opinion based on previous NBS model on SCID * [15,51]
Costs of symptomatic diagnosis	Carter et al. 2023 [52] Maggi et al. 2022 [53] NHS reference costs
Treatment costs (list prices)	Review of NICE appraisals [54]
Health state costs	Review of NICE appraisals [54]

* SCID: Severe Combined Immunodeficiency.

**Table 2:** Patients identified by SMA type and *SMN2* copy numbers.

**BSC arm**	**Patients diagnosed symptomatically**				
	73.17				
	**Patients by SMA type**				
	***SMA Type 0***	***SMA Type 1***	***SMA Type 2***	***SMA Type 3***	***SMA Type 4***
	0.53	40.32	20.16	9.04	3.12
**No NBS screening arm**	**Patients diagnosed via family history**				
	0.73				
	**Patients by *SMN2* copies**				
	***1 SMN2 copy***	***2 SMN2 copies***	***3 SMN2 copies***	***4 SMN2 copies***	***5 SMN2 copies***
	0.01	0.31	0.20	0.18	0.03
	**Patients diagnosed symptomatically**				
	72.44				
	**Patients by SMA type**				
	***SMA Type 0***	***SMA Type 1***	***SMA Type 2***	***SMA Type 3***	***SMA Type 4***
	0.53	39.91	19.96	8.95	3.09
**NBS screening arm**	**Patients diagnosed presymptomatically**				
	69.44				
	**Patients by *SMN2* copies**				
	***1 SMN2 copy***	***2 SMN2 copies***	***3 SMN2 copies***	***4 SMN2 copies***	***5 SMN2 copies***
	0.51	29.36	19.28	17.39	2.91
	**Patients diagnosed symptomatically**				
	3.73				
	**Patients by SMA type**				
	***SMA Type 0***	***SMA Type 1***	***SMA Type 2***	***SMA Type 3***	***SMA Type 4***
	0.03	2.05	1.03	0.46	0.16

Table 2 presents the proportions of the different SMA types (for those detected symptomatically) and *SMN2* copy numbers (for those detected presymptomatically) in the BSC arm, the No NBS screening arm, and the NBS screening arm. In the BSC arm, all patients are detected symptomatically. In the No NBS screening arm, almost all patients are detected symptomatically, and most of them have SMA Types 1 and 2. In the NBS screening arm, most patients are detected presymptomatically, and most of them have 2, 3, or 4 *SMN2* copy numbers. Abbreviations: BSC: best supportive care; NBS: newborn blood spot; SMA: spinal muscular atrophy; SMN: survival motor neuron.

**Table 3:** Cost-effectiveness results using all available treatments, using list prices and price discounts.

**All Available Treatments and Using List Prices**								
	**Screening/** **Diagnosis Costs**	**Treatment Costs**	**Health Care Costs**	**Total Costs**	**QALYs**	**LYs**	**Incremental Results**	
							**Cost/QALY Gained**	**Cost/LY Gained**
NBS screening	GBP 6,690,148	GBP 215,678,227	GBP 23,272,399	GBP 245,640,775	1432.71	1752.09	GBP 219,393	GBP 203,173
No NBS screening	GBP 182,628	GBP 194,282,221	GBP 56,875,837	GBP 251,340,686	845.75	1237.55	Dominated	Dominated
BSC	GBP 182,927	N/A	GBP 40,472,763	GBP 40,655,689	498.38	743.17	-	-
**All Available Treatments and Using Price Discounts**								
	**Screening/** **Diagnosis Costs**	**Treatment Costs**	**Health Care Costs**	**Total Costs**	**QALYs**	**LYs**	**Incremental Results**	
							**Cost/QALY Gained**	**Cost/LY Gained**
NBS screening	GBP 6,690,148	GBP 68,824,724	GBP 23,272,399	GBP 98,787,272	1432.71	1752.09	GBP 62,217	GBP 57,618
No NBS screening	GBP 182,628	GBP 56,868,003	GBP 56,875,837	GBP 113,926,468	845.75	1237.55	Dominated	Dominated
BSC	GBP 182,927	N/A	GBP 40,472,763	GBP 40,655,689	498.38	743.17	-	-

Table 3 presents the cost-effectiveness results of the base case analysis using all the treatments currently eligible and using list prices and treatments assuming price discounts. ICERs are calculated relative to the next least effective non-dominated option. NBS screening seems to be cost-saving and more effective compared to No NBS screening (i.e., No NBS screening is dominated). As such, the ICER for NBS screening is calculated compared to BSC. Abbreviations: BSC: best supportive care; ICER: incremental cost-effectiveness ratio; LY: life-year; GBP: Great British Pound; N/A: not applicable; NBS: newborn blood spot; QALY: quality-adjusted life year.

## Data Availability

The original contributions presented in this study are included in the article/Supplementary Material. Further inquiries can be directed to the corresponding author.

## Supplementary Materials

The following supporting information can be downloaded at: https://www.mdpi.com/article/10.3390/ijns12010003/s1. File S1: Model Inputs; File S2: Results of probabilistic sensitivity analysis.
