# Supplementary material for: Newborn Screening for Spinal Muscular Atrophy in the UK: Use of Modelling to Identify Priorities for Ongoing Evaluation
Source: Int J Neonatal Screen. 2026 Jan 13;12(1):3. doi: 10.3390/ijns12010003 (PMC12821460; doi:10.3390/ijns12010003)
Supplement: Supplementary file 1 [file IJNS-12-00003-s001.zip › IJNS-3896709-File S1.pdf]

Table of Contents

File S1: Model Inputs 2
Model Inputs for Epidemiology 2
Model Inputs for Treatments 4
Model Inputs for Outcomes 12
Model Inputs for Costs 14
References 19

File S1: Model Inputs

Model Inputs for Epidemiology

The epidemiology parameters include the incidence of SMA, proportions of genotypes, and mapping between genotypes and phenotypes.

Incidence

Data on the incidence of SMA was sourced from a recent systematic review[1] which provided data from several countries. The clinical experts suggested that patients from Western Europe were the closest to the UK population, as such, the data from Belgium and Germany were pooled to estimate the incidence of SMA as 1 in 8200 newborns.

Proportions of SMN2 copy numbers

The proportions of different *SMN2* copy numbers were sourced from published literature. As before, data on patients from northern Europe (Germany, Belgium and Norway) were used to estimate the proportions of different *SMN2* copy numbers as shown below in Table S1. Patients in the NBS screening arm were categorised by the number of *SMN2* copies (1, 2, 3, 4, 5+), and the 5+ *SMN2* copies category was considered separately to 4 *SMN2* copies category as there are no recommended pharmacological treatments for patients with 5+ *SMN2* copies.

Data from the US from a large cohort of patients, over 6 million screened babies and 425 confirmed cases of SMA, has recently been published which includes the birth prevalence and the distribution of *SMN2* copy numbers[2] With a birth prevalence of 1 in 14,694 and the distribution of *SMN2* copy number shown below this implies a lower incidence and a more severe disease distribution than the Northern European data. This data was included as a sensitivity analysis.

Table S1. Proportions of different *SMN2* copy numbers in patients with SMA

**Country**	**N**	**Proportions of different *SMN2* copy numbers**				
		**1**	**2**	**3**	**4**	**5+**
**Germany nationwide screening** [3]	46	2.00%	43.00%	28.00%	22.00%	4.00%
**Germany pilot projects** [4]	67	0.00%	46.00%	24.00%	26.00%	4.00%
**Belgium** [5]	9	0.00%	44.44%	33.33%	22.22%	0.00%
**Norway** [6]	10	0.00%	50.00%	30.00%	20.00%	0.00%
**Overall proportions**		0.73%	42.88%	27.76%	25.04%	3.59%
**US**[2]	240	5%	49%	33%	13%	

Mapping of genotypes to phenotypes

The mapping of genotypes to phenotypes (i.e. explicit modelling of the link between *SMN2* copy number and SMA type) was based on data from Calucho et al [7] as shown in Table S2. The Spanish only cohort was used for carrying out the initial mapping as the testing was conducted in the same laboratory and according to the same methodology. However, the Spanish only cohort did not differentiate between types 3 and 4. In order to calculate these proportions, a smaller subset of the international data was used and these proportions were then used to

adjust the Spanish only Type 3+ cohort. Given the small numbers of patients with 5+ *SMN2* copies and with SMA Type 4, it was assumed that all those with 5+ *SMN2* copies would be SMA Type 4.

Table S2. Relationship between *SMN2* copy number and SMA Types

***SMN2* Copy number**	**SMA Type 0**	**SMA Type 1**	**SMA Type 2**	**SMA Type 3**	**SMA Type 4**
**1**	100.00%	0.00%	0.00%	0.00%	0.00%
**2**	0.00%	88.00%	9.00%	2.77%	0.23%
**3**	0.00%	6.00%	57.00%	36.72%	0.28%
**4**	0.00%	0.00%	0.00%	88.40%	11.60%
**5+**	0.00%	0.00%	0.00%	0.00%	100.00%

Proportions used in the model

Based on data from Tables S1 and S2, the proportions of patients in the NBS screening arm and No NBS screening arm of the model can be estimated as below (Table S3). These proportions are presented in Table S3 along with estimates from the literature on SMA Type distribution. Kekou et al [8] reports on the phenotypes of 361 individuals genetically diagnosed with SMA in Greece over a 24-year period. Konig et al [9] collected data on the incidence of SMA in Germany from neuromuscular centres, genetic institutes and the German patient registries. Data on subtypes of SMA were collected only from neuromuscular centres and the German patient registry for 758 patients. This did not include patients with Type 0 or Type 4. Calucho et al [7] only included the index cases (unrelated) of SMA and the study was designed to assess the correlation between SMA type and *SMN2* copy number rather than an epidemiological study of SMA in Spain. All studies are likely to suffer from ascertainment bias with severe patients (Type 0 & 1) less likely to be included due to the high mortality in this cohort and less severe patients (Type 4) also less likely to be included due to potential for under or misdiagnosis in this group.

Table S3. Proportions of patients in different groups in the NBS screening arm and No NBS screening arm of the model

**NBS screening arm**	**1 *SMN2* copy**	**2 *SMN2* copies**	**3 *SMN2* copies**	**4 *SMN2* copies**	**5+ *SMN2* copies**
Proportions	0.73%	42.88%	27.76%	25.04%	3.59%
**No NBS screening arm**	**SMA Type 0**	**SMA Type 1**	**SMA Type 2**	**SMA Type 3**	**SMA Type 4**
Model Proportions	0.73%	55.10%	27.55%	12.35%	4.27%
Ogino et al [10]	N/R	58%	29%	13%	N/R
Calucho et al [7] Spain	N/R	43%	30%	27%	*
Kekou et al [8] Greece	2.5%	40%	26%	30%	1.5%
Konig et al[9] Germany	N/R	37%	41%	21%	N/R

*All walkers were grouped together as Type 3

Model Inputs for Treatments

Treatment mix

The reimbursement status of symptomatic treatments for SMA currently in the NHS and any restrictions on populations eligible for treatment are sourced from the NICE website. However, the elicitation of treatment mix was not constrained by reimbursement status of the treatments, given the status could change in the future. As such, expert opinion was solicited to specify the treatment mix describing the proportions of patients receiving the different treatments (Zolgensma, Spinraza, Risdiplam or BSC), either presymptomatically or symptomatically (based on the calculations in the screening module), according to the different *SMN2* copies (1, 2, 3, 4, 5+) in the NBS screening arm and SMA types (0, 1, 2, 3, 4) in the no NBS screening arm.

Table S4 shows the proportions of patients receiving the different treatments in the model, according to the number of *SMN2* copies (1, 2, 3, 4, 5+) in the NBS screening arm and SMA types (0, 1, 2, 3, 4) in the no NBS screening arm.

Table S4. Treatment mix in the NBS screening arm and No NBS screening arm

**NBS screening arm**	**1 *SMN2* copy**	**2 *SMN2* copies**	**3 *SMN2* copies**	**4 *SMN2* copies**	**5+ *SMN2* copies**
Nusinersen	0%	5%	5%	5%	0%
Zolgensma	0%	85%	85%	0%	0%
Risdiplam	0%	10%	10%	85%	0%
Best Supportive Care (BSC)*	100%	0%	0%	10%	100%
**No NBS screening arm**	**SMA Type 0**	**SMA Type 1**	**SMA Type 2**	**SMA Type 3**	**SMA Type 4**
Nusinersen	0%	2.5%	10%	10%	0%
Zolgensma	0%	85%	0%	0%	0%
Risdiplam	0%	7.5%	90%	90%	0%
Best Supportive Care (BSC)*	100%	5%	0%	0%	100%

*BSC in NBS screening arm refers to palliative style care for those with 1 *SMN2* copy and symptom management for those with 5+ copies, while in No NBS screening arm, BSC refers to palliative style care for those with SMA Type 1 and symptom management for those with SMA Types 3 & 4

Treatment in the NBS screening arm

In the NBS screening arm, the model uses 99.9% sensitivity and specificity for the initial PCR test, and assumes 100% specificity after confirmatory testing (as any false positives would be identified as not having SMA during the genetic testing). Note that the initial PCR test will not detect patients with other variants (i.e. the 5% of patients who do not have homozygous deletions in *SMN1*), as such the 99.9% sensitivity for the initial PCR test is for the rest of the patients.

Patients correctly identified in the NBS screening arm as having SMA (i.e. the true positives) would receive appropriate care with true positives receiving early and presymptomatic treatment, depending on the *SMN2* copy number. Patients with 1 *SMN2* copy would have Type 0 SMA and would receive palliative care as the prognosis is poor for these patients (death in the first month of life), without expectation of improvement with treatment. For patients with 2 *SMN2* copies, based on published literature, the average time taken for diagnosis and treatment is around 3 weeks. Around half of the patients with 2 *SMN2* copies would show symptoms by this time as shown in Table S5, and these patients would receive early symptomatic treatment. Clinical experts suggested that around 1-2% of new patients with 2 *SMN2* copies would show symptoms each day, and this would be used to perform scenario analyses with shorter diagnostic time interval of NBS (e.g. 2 weeks compared to the base case of 3 weeks), where more patients with 2 *SMN2* copies would receive presymptomatic treatment. All patients with 3 and 4 *SMN2* copies would be asymptomatic prior to treatment, as the symptom onset for type 2 and 3 SMA is around 6 months and 2.5 years, respectively. Patients with 5+ *SMN2* copies would be under annual follow-up and if symptomatic, would receive symptom management as patients with SMA Type 4.

Patients correctly identified in the NBS screening arm as not having SMA (i.e. true negatives) were assumed to be the same as general population and would not incur any further costs.

Patients with SMA missed during NBS screening (i.e. false negatives and compound heterozygotes) would receive treatment after symptom onset. Approximately 5% of patients with SMA are compound heterozygotes (deletion and point mutation) and a small proportion (0.1%) of patients are missed during NBS screening (due to error), and these patients would be identified symptomatically.

The small proportion of patients who were incorrectly identified as having SMA (i.e. false positives) during the initial PCR test were assumed to be identified as not having SMA during the genetic testing, which has 100% specificity as the gold standard.

Table S5 below describes the treatment timing based on the number of *SMN2* copies. Scenario analyses with shorter diagnostic time interval of NBS, where more patients with 2 *SMN2* copies would receive presymptomatic treatment were also performed.

Table S5. Treatment timing in the NBS screening arm based on number of *SMN2* copies

***SMN2* Copies**	**Presymptomatic treatment**	**Early Symptomatic treatment**	**Delayed symptomatic treatment**	**BSC***
1	-	-	-	100%
2	48%	52%	(False Negatives)	-
3	100%	0%	(False Negatives)	-
4	100%	0%	(False Negatives)	-
5+	-	-	-	100%

*BSC – palliative care for those with 1 *SMN2* copies and symptom management for those with 5+ *SMN2* copies

Treatment in the No NBS screening arm

In the No NBS screening arm, it was assumed that patients with family history of SMA would receive testing for SMA and would be detected pre-symptomatically. Some patients may be diagnosed at birth or shortly after without NBS screening due to a family history of the disease and would therefore benefit from early diagnosis and treatment. Discussion during the workshops indicated that this is relatively rare occurrence with most families opting for pre-implantation genetic testing or prenatal testing. It was suggested 1% of patients with SMA would be detected via family history and this estimate is used in the base case analysis.

The rest of the 99% of patients with SMA (i.e. those without family history of SMA) would receive treatment after developing symptoms. The average time of symptom onset (i.e. typical age at presentation) is used in the base case analyses as presented in Table S6 below.

Table S6. Age of symptom onset by SMA Type

SMA Type	Mean age at presentation (i.e. age of symptom onset)
0	At birth
1	3 months
2	12 months
3	2.5 years
4	>18 years

Effectiveness of presymptomatic treatment

The effectiveness of presymptomatic treatment is captured in terms of the motor function milestones achieved over different time points. This data is captured from the pivotal trials separately for each of the 3 treatments (Zolgensma, Spinraza and Risdiplam) based on the number of *SMN2* copies, are shown in Tables S7 and S8. It should be noted that the effectiveness data for presymptomatic treatment from pivotal trials was triangulated against other sources (e.g. registry data and other real world data), where available. In the model, the motor function milestones at the end of the study period are carried forward until month 36 (i.e. duration of the short-term period in the model).

As there is no data for the effectiveness of presymptomatic treatment for patients with 4 *SMN2* copies, it was assumed to be 100% for all motor function milestones (i.e. same as general population).

Table S7. Effectiveness of Presymptomatic Treatment for patients with 2 *SMN2* copies

**Zolgensma – based on SPRINT trial reported in supplementary material of Strauss et al 2022a**[11]<br>**Number of patients: 14**				
**Month**	**Not sitting**	**Sitting**	**Walking with assistance**	**Walking w/o assistance**
6	64.3%	35.7%	-	-
12	-	57.2%	35.7%	7.1%
18	-	14.3%	14.3%	71.4%
24	-	14.3%	14.3%	71.4%
**Risdiplam – based on RAINBOWFISH trial results reported in Servais et al 2024** [12]<br>**Number of patients: 5 (3 patients, not included, withdrawn to receive Zolgensma)**				
**Month**	**Not sitting**	**Sitting**	**Walking with assistance**	**Walking w/o assistance**
6	80%	20%	N/R	-
12	-	80%	N/R	20%

18	-	80%	N/R	20%
24	-	40%	N/R	60%
**Nusinersen – based on NURTURE trial reported in Crawford et al 2023** [13]<br>**Number of patients: 15**				
**Month**	**Not sitting**	**Sitting**	**Walking with assistance**	**Walking w/o assistance**
6	66.7%	33.3%	-	-
12	6.67%	73.3%	20%	-
18	-	40%	40%	20%
24	-	20%	20%	60%
30	-	20%	-	80%
36	-	13.3%	-	86.7%

*Note that those who were walking with assistance at the end of the trial follow-up were assigned to walking with assistance health state in the model, and those who were walking at the end of the trial follow-up were assigned to the broad range of normal development (BRND) health state in the model.

Table S8. Effectiveness of Presymptomatic Treatment for patients with 3 *SMN2* copies

**Zolgensma – based on SPRINT trial reported in supplementary material of Strauss et al 2022b** [14]<br>**Number of patients: 15**				
**Month**	**Not sitting**	**Sitting**	**Walking with assistance**	**Walking w/o assistance**
6	53.3%	46.7%	-	-
12	6.7%	20%	33.3%	40%
18	-	6.7%	-	93.3%
24	-	6.7%	-	93.3%
**Risdiplam – based on RAINBOWFISH trial results reported in Servais et al 2024** [12]<br>**Number of patients: 13**				
**Month**	**Not sitting**	**Sitting**	**Walking with assistance**	**Walking w/o assistance**
6	100%	0%	N/R	-
12	-	92%	N/R	8%

18	-	8%	N/R	92%
24	-	0%	N/R	100%
**Nusinersen – based on NURTURE trial reported in Crawford et al 2023** [13]<br>**Number of patients: 10**				
**Month**	**Not sitting**	**Sitting**	**Walking with assistance**	**Walking w/o assistance**
6	40%	60%	-	-
12	-	30%	10%	60%
18	-	-	-	100%
24	-	-	-	100%
30	-	-	-	100%
36	-	-	-	100%

* As there is no data for the effectiveness of presymptomatic treatment for patients with 4 *SMN2* copies, it was assumed to be 100% for all motor function milestones (i.e. the same as general population). Also, note that those who were walking with assistance at the end of the trial follow-up were assigned to walking with assistance health state in the model, and those who were walking at the end of the trial follow-up were assigned to the broad range of normal development (BRND) health state in the model.

Effectiveness of symptomatic treatment

The effectiveness of symptomatic treatment, in terms of the motor function milestones achieved over different time points, were captured from the pivotal trials separately for each of the 3 treatments (Zolgensma, Spinraza and Risdiplam) based on the type of SMA, as shown in Tables S9 and S10. The effectiveness data for symptomatic treatment from pivotal trials was triangulated against other sources (e.g. registry data such as SMA Reach and other real world data), where available.

Zolgensma is currently authorised in the UK for patients with SMA Type 1 only, and there is no data on treatment effectiveness of Zolgensma for Type 2 and Type 3 SMA. Given this, the model does not include the possibility of selecting Zolgensma for patients with Type 2 and Type 3 SMA.

Whilst risdiplam is authorised for patients with Type 2 and Type 3 SMA, there is no data on treatment effectiveness in terms of motor function milestones for these patients. As such, for patients with Type 2 and Type 3 SMA receiving risdiplam, the model will assume that the treatment effectiveness of risdiplam to be the same as that of nusinersen.

Table S9. Effectiveness of symptomatic treatment for patients with Type 1 SMA

**Zolgensma – pooled StriveEU & StriveUS (post 18 month based on improvement seen in START extension) Number of patients 55 (StriveEU & Strive US) 10 (START)**						
**Month**	**Dead**	**Permanent Ventilation**	**Not sitting**	**Sitting**	**Walking with assistance**	**Walking w/o assistance**
6	1.8%	-	98.2%	-	-	-
12	3.6%	3.6%	63.6%	27.3%	1.8%	-
18	3.6%	3.6%	38.2%	50.9%	-	3.6%
24	3.6%	3.6%	19.1%	70%	-	3.6%
30	3.6%	3.6%	19.1%	70%	-	3.6%
36	3.6%	3.6%	9.5%	79.5%	-	3.6%
**Risdiplam – based on FIREFISH trial reported in Masson et al 2022** [15] **and Deconinck et al 2022** [16] **(conference poster)*** **Number of patients: 41 (Masson et al 2022), 48 (Deconinck et al 2022)**						
**Month**	**Dead**	**Permanent Ventilation**	**Not sitting**	**Sitting**	**Walking with assistance**	**Walking w/o assistance**
12	7.3%	-	75.6%	17.1%	-	-
24	7.3%	2.4%	46.3%	43.9%	-	-
36	9%	10%	8.1%	66.7%	6.25%	-
**Nusinersen – based on data from SHINE Study, Castro et al** [17]						
**Month**	**Dead**	**Permanent Ventilation**	**Not sitting**	**Sitting**	**Walking with assistance**	**Walking w/o assistance**
6	13.9%	15.9%	66.3%	3.9%	-	-
12	17.3%	27.8%	42.4%	12.4%	-	-
18	22.1%	29.5%	25.7%	22.6%	-	-
24	22.1%	29.5%	29.6%	18.7%	-	-
30	22.1%	29.5%	29.6%	18.7%	-	-

***** Note that those who were walking with assistance at the end of the trial follow-up were assigned to walking with assistance health state in the model, and those who were walking at the end of the trial follow-up were assigned to the broad range of normal development (BRND) health state in the model.

Table S10. Effectiveness of symptomatic treatment with Nusinersen for patients with Type 2 SMA and Type 3 SMA

**Nusinersen for patients with Type 2 SMA – based on ENDEAR reported in Mercuri et al 2018** [18] **with longer follow up estimated from Darras et al 2019** [19]**; Number of patients: 84**				
**Month**	**Not sitting**	**Sitting**	**Walking with assistance**	**Walking w/o assistance**
6	-	100%	-	-
12	-	76.2%	23.8%	-
18	-	91.7%	8.3%	-
24	-	91.7%	8.3%	-
30	-	90.5%	9.5%	-
36	-	85.7%	9.5%	4.7%
**Nusinersen for patients with Type 3 SMA – based on Darras et al 2019** [19] **Number of patients: 17**				
**Month**	**Not sitting**	**Sitting**	**Walking with assistance**	**Walking w/o assistance**
6	-	100%	-	-
12	-	-	-	100%
18	-	-	-	100%
24	-	-	-	100%
30	-	11.8%	11.8%	78.5%
36	-	11.8%	-	88.2%

***** Note that those who were walking with assistance at the end of the trial follow-up were assigned to walking with assistance health state in the model, and those who were walking at the end of the trial follow-up were assigned to the broad range of normal development (BRND) health state in the model.

Incorporating treatment effectiveness data in the model

Each model health state (Permanent Ventilations (PV), Not Sitting, Sitting, Walking with Assistance, Broad Range of Normal Development (BRND)) has a cost and a utility value associated with it to account to the impact of SMA symptoms on the patient. As the model starts from birth, no babies will be sitting or walking at the beginning. However, these babies should not incur the costs and lower utility value associated with the Not Sitting health state as it is part of normal development that they would be not sitting at this age.

In order to account for this, the normal developmental rules as shown in Figure S1 were used to adjust the milestones reported in the pivotal trials. One model cycle (6 months) was allowed over the normal World Health

Organisation (WHO) developmental window to account for delays between a child achieving a milestone and their next trial assessment. Some additional assumptions based on the SMA Type or number of *SMN2* copy were also used.

Figure S1. Normal Development Rules and the order applied

**Rule**	**Starting Health State**	**Exceptions**
1. If they have achieved Walking with Assistance (18 months) or Walking without assistance (24 months)	BRND	No patients with Type 1 SMA start in the BRND health state as they would need to show delayed milestones to order to present symptomatically
2. If only sitting at 18 months	Sitting	Does not apply to Type 2 SMA as assume normal development at 6 months and begin to lose milestones at 12 months
3. If not sitting at 12 months	Not sitting	If **all** patients eventually sit, no patients start in the Not Sitting health state

Model Inputs for Outcomes

Long-term survival modelling

For the long-term modelling, in line with the feedback from previous NICE appraisals, the model assumes that the patients cannot transition to better motor function milestones beyond the study follow-up period. Based on expert opinion, the scenario analyses include assumptions around whether the patients continue to stay in the same motor function milestones or whether there is treatment waning (i.e. some patients lose their motor function milestones in the long-term).

The mortality risks, based on the health states (i.e. motor function milestones), are sourced from key literature such as the NICE appraisals and published systematic reviews of cost-effectiveness studies of SMA. The mortality in the long-term is modelled using survival curves for each of the motor function milestones, and the model includes hazard ratios to estimate the impact of assuming better or worse survival in scenario analyses.

Table S11. Survival by motor function milestones on treatment

Parameter	Mean survival	Sources
Permanent ventilation	5 years*	Gregoretti et al [20]
Not Sitting	5 years*	
Sitting	30 years	Zerres and Schoneborn et al [21]
Walking with assistance	70.90 years	Assumption
Broad range of normal development	78.69 years	General population mortality - Assumption

*assumed to be 2 years for patients on BSC

Utilities

Patient utilities were sourced from the published systematic reviews of utilities in SMA and triangulated with expert input and feedback from NICE appraisals. Caregiver disutilities (for false positive cases in the NBS screening arm as well as longer term disutilities caring for patients with SMA) were captured from published systematic reviews of utilities in SMA and expert input.

The utilities for the different health states (based on motor function milestones) used in the base case analyses are derived from different sources, as presented in Table S12. The utilities from Bastida et al are used for the 'permanent ventilation' and 'not sitting' health states, which were assumed to have same utility values. The utility for 'sitting' health state is captured from Tappenden et al. The utilities for 'Walking with assistance' and 'BRND' health states are captured from the mapped values to Pediatric Quality of Life Inventory (PedSQL) data from CHERISH trial, as the patients in the mapping study were all healthy patients. As such, the utilities used in the base case are a combination of different sources.

Table S12. Utilities used in base case analyses

Health State	Utility	Source
Permanent ventilation	0.19	López-Bastida et al [22]
Not sitting	0.19	
Sitting	0.60	Tappenden et al [23]
Walking with assistance	0.80	Assumption
BRND	0.9	Assumption

Scenario analyses were also performed using utility data from other published literature as shown in Table S13 below.

Table S13. Utilities used in scenario analyses

**Health State**	**Utilities from Landfelt et al** [24] **(using EQ-5D-5L)**
Permanent ventilation	0.19
Not sitting	0.26
Sitting	0.46
Walking with assistance	0.76
Normal function	General population utility

Model Inputs for Costs

Costs of NBS screening
The resources required for setting up the NBS including laboratory adaptations/equipment, programme and pathway adaptations, cost per baby screened were sourced from previous UK NSC evaluations,[25,26] such as Severe Combined Immunodeficiency (SCID) screening, where available, and supplemented with expert opinion.

The cost of setting up and running the facilities to test the DBS using ddPCR was estimated via expert opinion and costs from the proposed SMA NBS in-service evaluation (ISE). It has an estimated costs of £75,400 per annum including £24,000 for equipment and maintenance costs, £49,000 for staff costs, and £2,400 for reagent costs (personal communication – email 28/11/24). The cost per baby screened will depend on the cohort size, incidence and test characteristics of the PCR test. Assuming 600,000 births per annum (pa) results in a total of 146 tests per annum (i.e. 73 tests per annum on DBS samples + 73 tests pa on liquid blood). Assuming a 10-year amortisation period results in cost of £510 per test (i.e. 75000/120). However, a conservative estimate of £700 per test was used in the model.

Transportation costs for samples has been estimated at £100 per sample based on expert opinion. The time to the clinical service of organising a referral and seeing the patient was estimated via expert opinion and costed using NHS National Costs Collection or the Unit Costs of Health and Social Care.

Table S14. Costs of screening and confirmatory testing

	Cost	Included costs	Source
qRT-PCR test (DBS)	£7	• Test kit<br>• Staff<br>• Equipment<br>• Laboratory adaptations<br>• Consumables<br>• Quality assurance<br>• IT changes	SCID screening evaluation and expert opinion
ddPCR (DBS and venous blood sample)	£700 per test	• Equipment & maintenance costs<br>• Staff costs<br>• Reagent costs	Expert opinion – proposed SMA screening in-service evaluation (ISE)
Sample transportation	£100	Rapid sample transportation cost	Literature and assumption
Referral to clinician	3x outpatient paediatric neurology<br>Total £1392	Staff costs – referral, outpatient appointment, phlebotomy	Expert opinion and routine data sources

DBS: Dried blood spot, ddPCR: Digital droplet polymerase chain reaction, qRT-PCR: quantitative reverse transcription polymerase chain reaction, SCID: Severe combined immunodeficiency

Costs of symptomatic diagnosis

As shown in Table S15 for patients diagnosed symptomatically, the diagnostic costs were estimated from the literature. A study of Irish patients found they had had on average 5 contacts with health services prior to the diagnosis. This was costed as 5 GP appointments. The number of diagnostic tests is based on an Italian study that estimated the number of diagnostic tests that patients underwent prior to genetic testing. And in line with screening arm, it was assumed that patients would have 2 to 3 neurology appointments for their ddPCR or MRI test and results. A total cost of £2500 for the diagnostic odyssey of patients detected symptomatically was used.

Table S15. Costs of diagnosis in symptomatic patients

Health resource used	Number	Cost	Total	Source
Health visits prior to diagnosis (GP appointment)	5	£49	£245	Carter et al [27]
MRI	1	£309	£309	Maggi et al [28]
Outpatient paediatric neurology	2.5	£464	£1,160	Expert opinion and routine data sources
ddPCR	1	£700	£700	Calculation

Treatment costs

The costs of treatments are under confidential patient access schemes, and without access to this confidential pricing data, the list prices for the treatment costs were used in the base case analyses. These are summarised in Table S16.

However, sensitivity analyses were performed using discounts of 30% for Zolgensma, and 90% for the other 2 drugs to understand the impact on cost-effectiveness. Zolgensma is a one-off treatment, so a lower discount was assumed, while the other 2 drugs have to be administered through the patient's lifetime, so greater discount was assumed.

Table S16. List prices of the treatments

	List price per unit	Dosage	Administration costs	Total annual cost per patient
Nusinersen	£75,000 per vial	Four loading doses (days 0, 14, 28, and 63), and afterwards every 4 months	£700 to £1600 depending on age	£456,500 in year and £227,500 for subsequent years
Zolgensma	£1,795,000	one-time, single-dose of 1.1x1014 vg/kg by IV infusion	£3,500	£1,798,500 (lifetime cost)
Risdiplam	£7,900 per 60-mg (80-ml) vial	if less than 20kg, a vial approximately every 44 days Otherwise, every 12 days	90% of patients would receive via homecare and costs borne by the company	£65,500 if less than 20kg £240,450 otherwise

Nusinersen has a list price of £75,000 per vial and is administered via repeated intrathecal injections. Patients are given four loading doses (days 0, 14, 28, and 63) and thereafter are treated every 4 months for life, resulting in a total annual treatment cost of £450,000 for the first year and £225,000 for subsequent years at the list price. In their submission to NICE, the company assumed 40% of all nusinersen administrations are in an inpatient setting, 30% are in an outpatient setting and the remaining 30% are in a day case setting. The costs for lumbar puncture were taken from NHS Reference Costs using HRG codes HC72A (Diagnostic Spinal Puncture, 19 years and over), HC72B (Diagnostic Spinal Puncture, between 6 and 18 years) and HC72C (Diagnostic Spinal Puncture, 5 years and under). The company calculated weighted mean administration costs of approximately £1,600 for patients aged 5 years and under, £1,450 for those aged between 6 and 18 years and £700 for those aged 19 years and over.

Zolgensma has a list price of £1,795,000 and is administered as one-time, single-dose by intravenous (IV) infusion over approximately 60 minutes at a dose of 1.1×10^{14} vector genomes/kilogram. The administration cost is £3,500 based on the NHS Schedule of Reference Costs, using weighted average of codes relating paediatric nervous system disorders and cerebral degenerations or miscellaneous disorders of nervous system (EL- PR01A-E and EL - AA25C-G).

Risdiplam has a list price of £7,900 per 60-mg (80-ml) vial and the dosage for Risdiplam is age and weight dependent: in infants < 2 months, 0.15 mg/kg once a day, in subjects 2 months to 2 years, 0.2 mg/kg, once a day, in those > 2 years and up to 19 kg, 0.25 mg/kg once a day, and in those > 2 years and over 20 kg, 5 mg once a day. In the model, this was implemented as follows: for infants under the age of 2, on average 5.48 vials were used over 6-months, for 2-6 year olds, on average 11.41 vials were used over 6 months, and for those greater than 6 years, the full dose was used which resulted in an average of 15.22 vials over the 6-month period. This assumption was based on risdiplam dosing and data on the average weight by age suggested by WHO. Also, it was assumed that the majority of patients will receive risdiplam via homecare, the cost for which will be covered by Roche. However, 10% of patients were assumed to choose to have risdiplam administered through the hospital instead of home delivery, and it was assumed that 5 minutes of pharmacist time (cost: £44 per hour) will be required to reconstitute one vial of risdiplam. This resulted in an administrative cost of £66 per 6-month period.

Health state costs

The health state costs were sourced from previous NICE appraisals, and the caregiver and productivity costs were sourced from key published literature including systematic reviews of costs/cost-effectiveness of screening for SMA and review of economic modelling evidence of NICE appraisals.[29]

There is some variation in the health state costs suggested by the companies in their NICE submissions, as noted in Table S17 below. Based on the expert input from the workshops, the model used the costs in the Nusinersen NICE submission but has added additional costs for the Broad Range of Normal Development health state to account for appointments and tests in this population: these have been estimated at £1,000 per cycle for an annual cost of £2,000 per year.

Table S17. Annual costs by health state in the company submissions to NICE

**Health State**	**Annual Costs in Nusinersen NICE submission**	**Costs in Zolgensma NICE submission**	**Costs in Risdiplam NICE submission**	**Costs used in the model**
Permanent ventilation	£259,371	£283,710	£259,368	£259,330
Not sitting	£148,214	£112,500	£148,212	£148,214
Sitting	£68,322	£67,567	£108,276	£68,312
Walking with assistance	£20,229	£8,333	£21,768	£21,768
BRND	N/A	£8,333	N/A	£2,000

References

1. Dangouloff, T.; Botty, C.; Beaudart, C.; Servais, L.; Hiligsmann, M. Systematic Literature Review of the Economic Burden of Spinal Muscular Atrophy and Economic Evaluations of Treatments. *Orphanet Journal Of Rare Diseases* **01 23**, *16*, 47, doi:10.1186/s13023-021-01695-7.
2. Belter, L.; Taylor, J.L.; Jorgensen, E.; Glascock, J.; Whitmire, S.M.; Tingey, J.J.; Schroth, M. Newborn Screening and Birth Prevalence for Spinal Muscular Atrophy in the US. *JAMA Pediatrics* **2024**, *178*, 946–949, doi:10.1001/jamapediatrics.2024.1911.
3. Muller-Felber, W.; Blaschek, A.; Schwartz, O.; Glaser, D.; Nennstiel, U.; Brockow, I.; Wirth, B.; Burggraf, S.; Roschinger, W.; Becker, M.; et al. Newbornscreening SMA - From Pilot Project to Nationwide Screening in Germany. *Journal of neuromuscular diseases* **2023**, *10*, 55–65, doi:10/gs5qrq.
4. Vill, K.; Schwartz, O.; Blaschek, A.; Glaser, D.; Nennstiel, U.; Wirth, B.; Burggraf, S.; Roschinger, W.; Becker, M.; Czibere, L.; et al. Newborn Screening for Spinal Muscular Atrophy in Germany: Clinical Results after 2 Years. *Orphanet Journal Of Rare Diseases* **2021**, *16*, 153, doi:10.1186/s13023-021-01783-8.
5. Boemer, F.; Caberg, J.H.; Beckers, P.; Dideberg, V.; di Fiore, S.; Bours, V.; Marie, S.; Dewulf, J.; Marcelis, L.; Deconinck, N.; et al. Three Years Pilot of Spinal Muscular Atrophy Newborn Screening Turned into Official Program in Southern Belgium. *Scientific Reports* **2021**, *11*, 19922, doi:10.1038/s41598-021-99496-2.
6. Wallace, S.; Orstavik, K.; Rowe, A.; Strand, J. National Newborn Screening for SMA in Norway. *Neuromuscular Disorders* **2023**, *33*, S90, doi:10.1016/j.nmd.2023.07.102.
7. Calucho, M.; Bernal, S.; Alias, L.; March, F.; Vencesla, A.; Rodriguez-Alvarez, F.J.; Aller, E.; Fernandez, R.M.; Borrego, S.; Millan, J.M.; et al. Correlation between SMA Type and SMN2 Copy Number Revisited: An Analysis of 625 Unrelated Spanish Patients and a Compilation of 2834 Reported Cases. *Neuromuscular Disorders* **3AD**, *28*, 208–215, doi:10.1016/j.nmd.2018.01.003.
8. Kekou, K.; Svingou, M.; Sofocleous, C.; Mourtzi, N.; Nitsa, E.; Konstantinidis, G.; Youroukos, S.; Skiadas, K.; Katsalouli, M.; Pons, R.; et al. Evaluation of Genotypes and Epidemiology of Spinal Muscular Atrophy in Greece: A Nationwide Study Spanning 24 Years. *Journal of neuromuscular diseases* **2020**, *7*, 247–256, doi:10.3233/JND-190466.
9. Konig, K.; Pechmann, A.; Thiele, S.; Walter, M.C.; Schorling, D.; Tassoni, A.; Lochmuller, H.; Muller-Reible, C.; Kirschner, J. De-Duplicating Patient Records from Three Independent Data Sources Reveals the Incidence of Rare Neuromuscular Disorders in Germany. *Orphanet Journal Of Rare Diseases* **06 24**, *14*, 152, doi:10.1186/s13023-019-1125-2.
10. Ogino, S.; Wilson, R.B. Spinal Muscular Atrophy: Molecular Genetics and Diagnostics. *Expert Review of Molecular Diagnostics* **2004**, *4*, 15–29, doi:10.1586/14737159.4.1.15.
11. Strauss, K.A.; Farrar, M.A.; Muntoni, F.; Saito, K.; Mendell, J.R.; Servais, L.; McMillan, H.J.; Finkel, R.S.; Swoboda, K.J.; Kwon, J.M.; et al. Onasemnogene Abeparvovec for Presymptomatic Infants with Two Copies of SMN2 at Risk for Spinal Muscular Atrophy Type 1: The Phase III SPR1NT Trial. *Nat Med* **2022**, *28*, 1381–1389, doi:10.1038/s41591-022-01866-4.
12. Servais, L.; Finkel, R.; Farrar, M.; Vlodavets, D.; Zanoteli, E.; Al-Muhaizea, M.; Araújo, A.; Nelson, L.; Jaber, B.; Gorni, K.; et al. 21O RAINBOWFISH: 2-Year Efficacy and Safety Data of Risdiplam in Infants with Presymptomatic SMA. *Neuromuscular Disorders* **2024**, *43*, 104441.738, doi:10.1016/j.nmd.2024.07.747.
13. Crawford, T.O.; Swoboda, K.J.; De Vivo, D.C.; Bertini, E.; Hwu, W.L.; Finkel, R.S.; Kirschner, J.; Kuntz, N.L.; Nazario, A.N.; Parsons, J.A.; et al. Continued Benefit of Nusinersen Initiated in the Presymptomatic Stage of Spinal Muscular Atrophy: 5-Year Update of the NURTURE Study. *Muscle and Nerve* **2023**, *68*, 157–170, doi:10.1002/mus.27853.
14. Strauss, K.A.; Farrar, M.A.; Muntoni, F.; Saito, K.; Mendell, J.R.; Servais, L.; McMillan, H.J.; Finkel, R.S.; Swoboda, K.J.; Kwon, J.M.; et al. Onasemnogene Abeparvovec for Presymptomatic Infants with Three Copies of SMN2 at Risk for Spinal Muscular Atrophy: The Phase III SPR1NT Trial. *Nature Medicine* **2022**, *28*, 1390–1397, doi:10.1038/s41591-022-01867-3.
15. Masson, R.; Boespflug-Tanguy, O.; Darras, B.T.; Day, J.W.; Deconinck, N.; Klein, A.; Mazurkiewicz-Bedziska, M.; Mercuri, E.; Rose, K.; Servais, L.; et al. FIREFISH Parts 1 and 2: 24-Month Safety and Efficacy of Risdiplam in Type 1 SMA. *European Journal of Neurology* **2021**, *28(SUPPL 1)*, 395–396, doi:10.1111/ene.14974.
16. Deconinck, N.; Baranello, G.; Boespflug-Tanguy, O.; Day, J.; Klein, A.; Masson, R.; Mazurkiewicz-Beldzinska, M.; Mercuri, E.; Rose, K.; Servais, L.; et al. FIREFISH Parts 1 and 2: 36-Month Safety and Efficacy of Risdiplam in Type 1 Spinal Muscular Atrophy. *European Journal of Neurology* **2022**, *29(Supplement 1)*, 279, doi:10.1111/ene.15465.

17. Castro, D.; Finkel, R.S.; Farrar, M.A.; Tulinius, M.; Krosschell, K.J.; Saito, K.; Gambino, G.; Foster, R.; Bhan, I.; Wong, J.; et al. Nusinersen in Infantile-Onset Spinal Muscular Atrophy: Results from Longer-Term Treatment from the Open-Label Shine Extension Study. *Neurology. Conference: 72nd Annual Meeting of the American Academy of Neurology, AAN* **2020**, *94*.
18. Mercuri, E.; Darras, B.T.; Chiriboga, C.A.; Day, J.W.; Campbell, C.; Connolly, A.M.; Iannaccone, S.T.; Kirschner, J.; Kuntz, N.L.; Saito, K.; et al. Nusinersen versus Sham Control in Later-Onset Spinal Muscular Atrophy. *New England Journal of Medicine* **2018**, *378*, 625–635, doi:10.1056/NEJMoa1710504.
19. Darras, B.T.; Farrar, M.A.; Mercuri, E.; Finkel, R.S.; Foster, R.; Hughes, S.G.; Bhan, I.; Farwell, W.; Gheuens, S. An Integrated Safety Analysis of Infants and Children with Symptomatic Spinal Muscular Atrophy (SMA) Treated with Nusinersen in Seven Clinical Trials. *CNS Drugs* **9AD**, *33*, 919–932, doi:10.1007/s40263-019-00656-w.
20. Gregoretti, C.; Ottonello, G.; Chiarini Testa, M.B.; Mastella, C.; Ravà, L.; Bignamini, E.; Veljkovic, A.; Cutrera, R. Survival of Patients With Spinal Muscular Atrophy Type 1. *Pediatrics* **2013**, *131*, e1509–e1514, doi:10.1542/peds.2012-2278.
21. Zerres, K.; Wirth, B.; Rudnik-Schoneborn, S. Spinal Muscular Atrophy - Clinical and Genetic Correlations. *Neuromuscular Disorders* **1997**, *7(3)*, 202–207, doi:10.1016/S0960-8966%2897%2900459-8.
22. Lopez-Bastida, J.; Pena-Longobardo, L.M.; Aranda-Reneo, I.; Tizzano, E.; Sefton, M.; Oliva-Moreno, J. Social/Economic Costs and Health-Related Quality of Life in Patients with Spinal Muscular Atrophy (SMA) in Spain. *Orphanet Journal Of Rare Diseases* **08 18**, *12*, 141, doi:10.1186/s13023-017-0695-0.
23. Tappenden, P.; Hamilton, J.; Kaltenthaler, E.; Hock, E.; Rawdin, A.; Mukuria, C. *Nusinersen for Treating Spinal Muscular Atrophy: A Single Technology Appraisal*; School of Health and Related Research (ScHARR), 2018;
24. Landfeldt, E.; Edstrom, J.; Sejersen, T.; Tulinius, M.; Lochmuller, H.; Kirschner, J. Quality of Life of Patients with Spinal Muscular Atrophy: A Systematic Review. *European Journal of Paediatric Neurology* **2019**, *23*, 347–356, doi:10.1016/j.ejpn.2019.03.004.
25. Bessey, A.; Chilcott, J.; Leaviss, J.; de la Cruz, C.; Wong, R. A Cost-Effectiveness Analysis of Newborn Screening for Severe Combined Immunodeficiency in the UK. *International Journal of Neonatal Screening* **2019**, *5*, doi:10.3390/ijns5030028.
26. UK National Screening Committee (UK NSC) Newborn Screening Programme SCID Available online: https://view-health-screening-recommendations.service.gov.uk/scid/.
27. Carter, M.; Tobin, A.; Coy, L.; McDonald, D.; Hennessy, M.; O'Rourke, D. Room to Improve: The Diagnostic Journey of Spinal Muscular Atrophy. *European Journal of Paediatric Neurology* **2023**, *42*, 42–46, doi:10/gs5qq8.
28. Maggi, L.; Vita, G.; Marconi, E.; Taddeo, D.; Davi, M.; Lovato, V.; Cricelli, C.; Lapi, F. Opportunities for an Early Recognition of Spinal Muscular Atrophy in Primary Care: A Nationwide, Population-Based, Study in Italy. *Family Practice* **2022**, *11*, 11, doi:10.1093/fampra/cmac091.
29. Wiedmann, L.; Cairns, J. Review of Economic Modeling Evidence from NICE Appraisals of Rare Disease Treatments for Spinal Muscular Atrophy. *Expert Review of Pharmacoeconomics & Outcomes Research* **2023**, *23*, 469–482, doi:10.1080/14737167.2023.2193690.
