# Supplementary material for: Newborn Screening for Spinal Muscular Atrophy in the UK: Use of Modelling to Identify Priorities for Ongoing Evaluation
Source: Int J Neonatal Screen. 2026 Jan 13;12(1):3. doi: 10.3390/ijns12010003 (PMC12821460; doi:10.3390/ijns12010003)

Table of contents

File S2 - Results of probabilistic sensitivity analysis 2

Probabilistic results of base case analysis using all available treatments and list prices 2

Probabilistic results of base case analysis using all available treatments and price discounts 3

File S2 - Results of probabilistic sensitivity analysis

The results presented in the following section include the effects of accounting for uncertainty in the model parameters (the costs, utilities, and other parameters), characterised as probability distributions. Probabilistic sensitivity analysis (PSA) is undertaken whereby the model is rerun (1000 times), each time with a different value for the parameters, which are sampled from the probability distributions.

The scatterplot of the cost-effectiveness plane shows the incremental costs (y-axis) and incremental QALYs (x-axis) for each of the PSA runs. In this chart, if a model run for NBS screening had exactly the same costs and QALYs as No NBS screening then the 'sample' for that model run would appear at the origin. Samples plotted to the right of the y-axis have more QALYs than No NBS screening and samples plotted above the x-axis have more costs.

The cost-effectiveness acceptability curve (CEAC) shows the proportion of model runs for which each strategy is cost-effective over a range of potential willingness-to-pay thresholds.

Probabilistic results of base case analysis using all available treatments and list prices

Figure S1 presents the scatterplot of the cost-effectiveness plane, which shows the incremental costs (y-axis) and incremental QALYs (x-axis) for each of the PSA runs.

As the model is rerun 1000 times, each time with a different value for the parameters sampled from the probability distribution, in some of the sampled model runs NBS screening is more costly than No NBS screening. Also, whilst there is uncertainty in the magnitude of incremental QALYs, NBS screening is always more effective than No NBS screening (i.e. NBS screening always has higher QALYs compared to No NBS screening).

Figure S1. Scatterplot of incremental costs and QALYs of NBS compared to No NBS (analysis using all avail-able treatments and list prices)

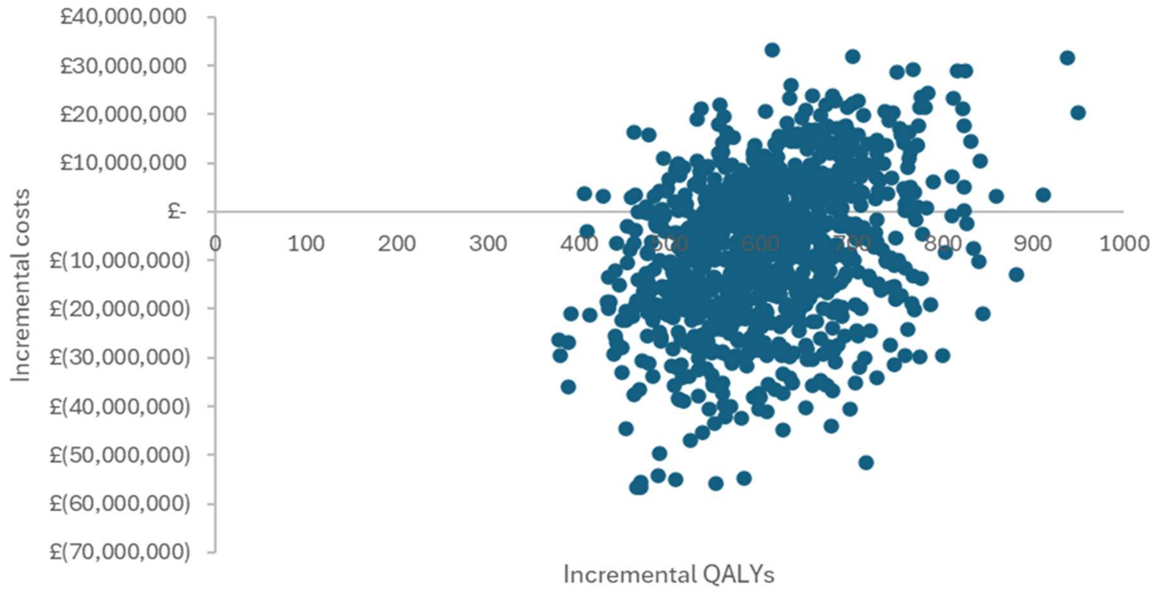

The cost-effectiveness acceptability curve (CEAC) in Figure S2 shows the proportion of model runs for which NBS screening is cost-effective over a range of potential willingness-to-pay thresholds. At a threshold of

£20,000/QALY, the percentage of model runs in which NBS screening was the most cost-effective strategy was around 90%, suggesting a 90% probability of NBS being cost-effective.

Figure S2. Probability of NBS being cost-effective at different thresholds (analysis using all available treatments and list prices)

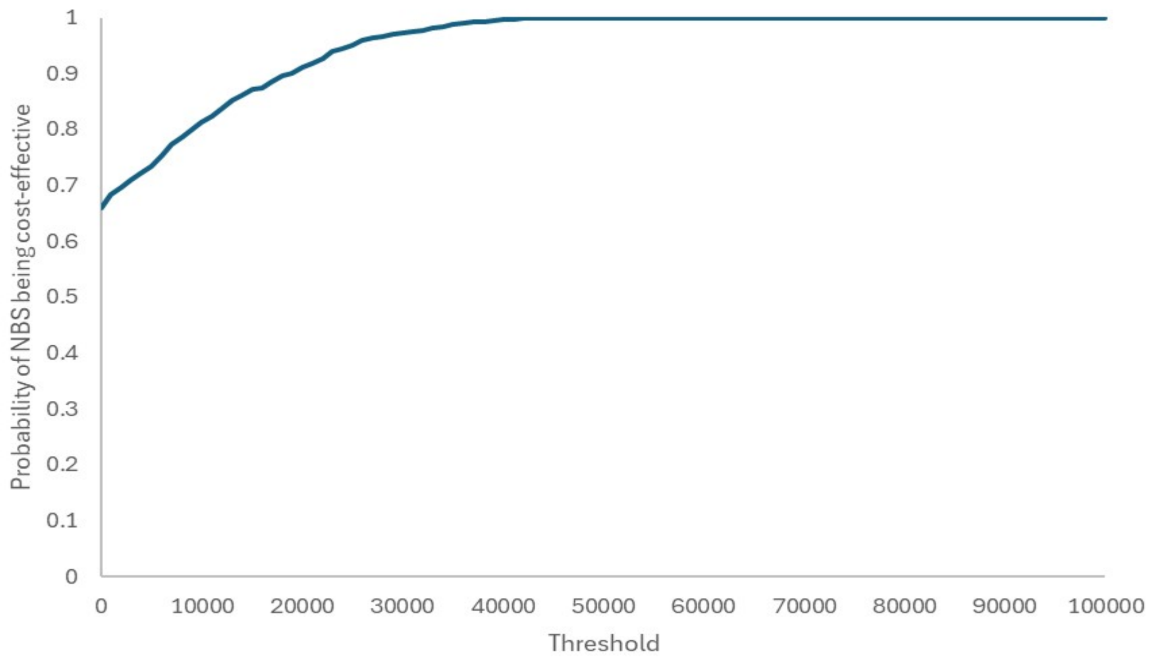

Probabilistic results of base case analysis using all available treatments and price discounts

In this analysis using price discounts, almost all of the sampled model runs in Figure S3 are below x-axis suggesting that NBS screening is less costly and more effective than No NBS screening. This can also be observed in Figure S4 which suggests 100% probability of NBS screening being cost-effective at thresholds greater than £20,000/QALY.

Figure S3. Scatterplot of incremental costs and QALYs of NBS compared to No NBS (analysis using all available treatments and price discounts)

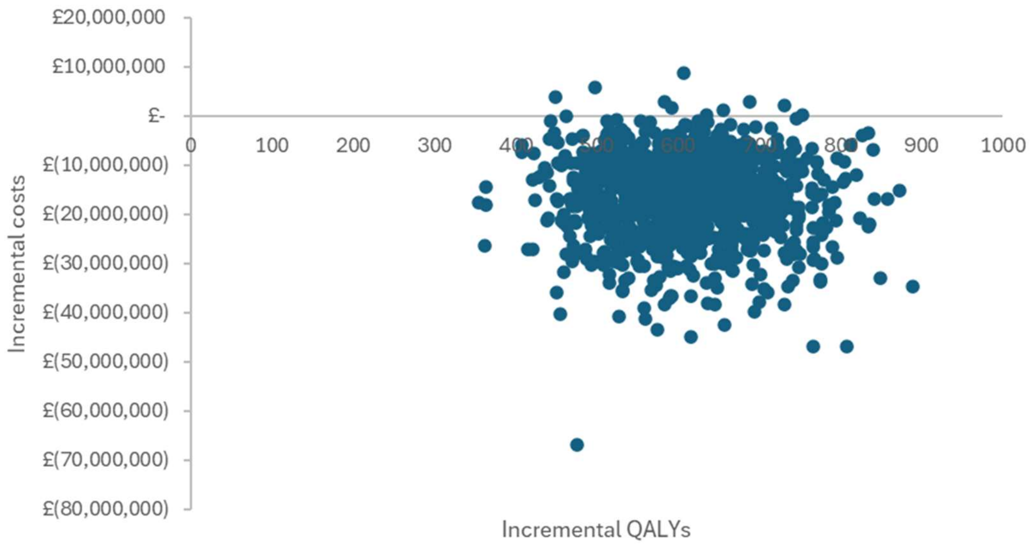

Figure S4. Probability of NBS being cost-effective at different thresholds (analysis using all available treatments and with price discounts)

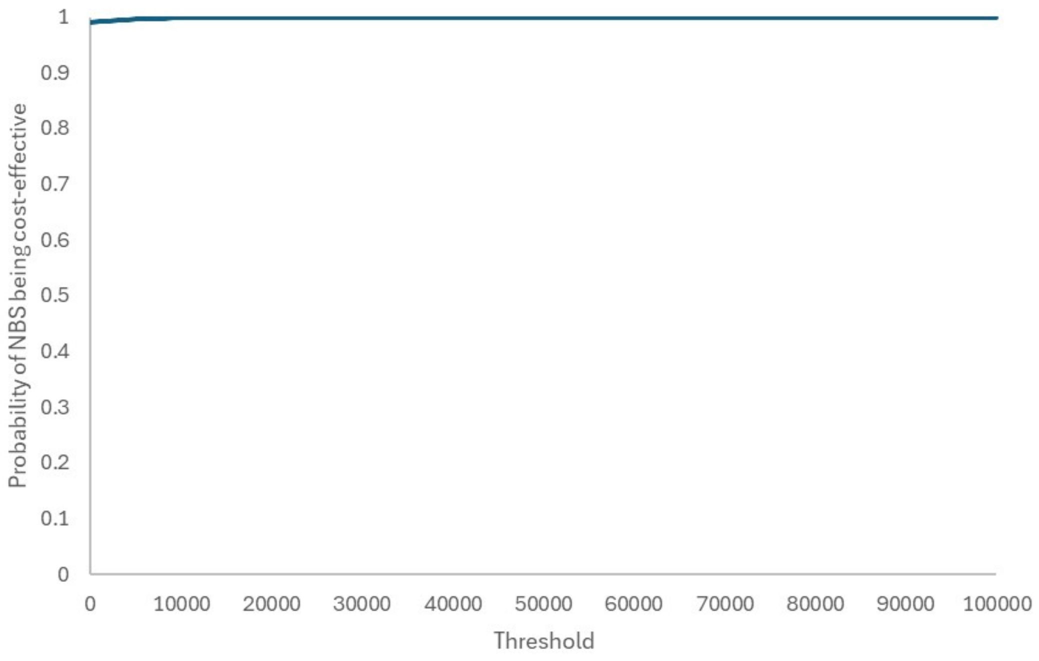

Supplement: Supplementary file 1 [file IJNS-12-00003-s001.zip › IJNS-3896709-File S2.pdf]
